# Energy Absorption and Beam Damage during Microfocus Synchrotron X-ray Diffraction

**DOI:** 10.1021/acs.jpclett.4c00497

**Published:** 2024-06-07

**Authors:** Štefan T. Stanko, Jürgen E. K. Schawe, Florian Spieckermann, Jürgen Eckert, Jörg F. Löffler

**Affiliations:** † Laboratory of Metal Physics and Technology, Department of Materials, ETH Zurich, 8093 Zurich, Switzerland; ‡ Mettler-Toledo GmbH, Analytical, 8606 Nänikon, Switzerland; § Department of Materials Science Chair of Materials Physics, Montanuniversität Leoben, 8700 Leoben, Austria; ∥ Erich Schmid Institute of Materials Science, Austrian Academy of Sciences, 8700 Leoben, Austria

## Abstract

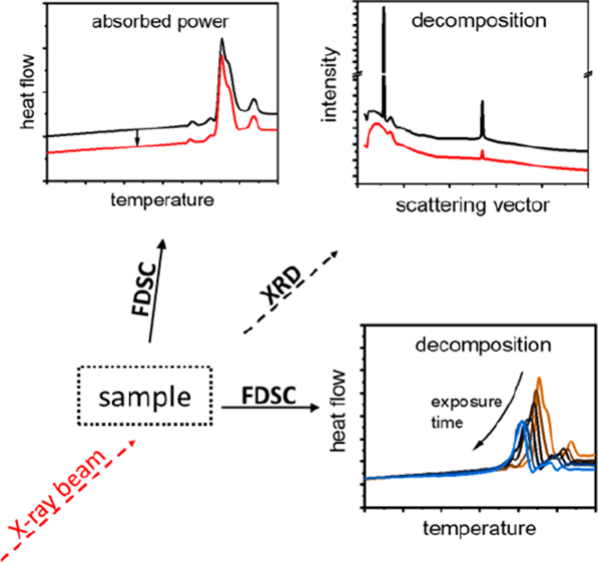

In this study, we combine *in situ* fast differential scanning calorimetry (FDSC) with synchrotron X-ray measurements to study simultaneously the structure and thermophysical properties of materials. Using the example of the organic compound BCH-52, we show that the X-ray beam can heat the sample and induce a shift of the heat-flow signal. The aim of this paper is to investigate the influence of radiation on sample behavior. The calorimetric data is used to quantify the absorbed beam energy and, together with the diffraction data, reveal an irreversible damage of the sample. The results are especially important for materials with high absorption coefficients and for high-energy X-ray and electron beams. Our findings illustrate that FDSC combined with X-ray diffraction is a suitable characterization method when beam damage must be minimized.

High-intensity and high-energy micro- and nanofocus X-ray sources using detectors with high acquisition rate enable high-resolution diffraction experiments at various conditions.^1−3^ This has initiated fundamental progress in the analysis of metastable structures and phase transformations of materials. A significant drawback of high-brilliance X-ray sources for materials characterization is, however, a potential heating of the sample^4,5^ and irreversible radiation damage.^6−9^ The changes in the original structure will lead to difficulties in *in situ* measurements and reproducing X-ray experiments.^10^ This is not only relevant to solid organic materials but also to aqueous solutions^11^ and inorganic samples, where beam heating might trigger irreversible processes such as changes of phase composition, especially if metastable phases are present. These effects may, however, be detected by fast differential scanning calorimetry (FDSC).

A combination of X-ray diffraction (XRD) and conventional differential scanning calorimetry (DSC) or MEMS chip-based FDSC^12^ enables the simultaneous characterization of structural and thermophysical properties, and thus allows to determine phase stability and transformation kinetics. This is of particular interest for metastable systems, such as polymorphic organic compounds and polymers^13,14^ or bulk metallic glass (BMG)-forming alloys.^15−17^ Attempts to characterize the stability of polymeric phases by wide-angle X-ray diffraction (WAXD) have generally been performed on samples that were solidified by *ex situ* cooling using dedicated devices^18,19^ or FDSC.^20^*In situ* X-ray heating experiments at rates of up to 1000 K s^–1^ were conducted by Orava et al.,^21^ following a real-time WAXD analysis.^22−24^ Additional heat-flow information has been obtained by the modification of a standard DSC furnace and crucible, in order to integrate it in a synchrotron beamline to characterize organic compounds^25,26^ and metallic alloys.^27^

Initially, the challenge in simultaneously using XRD and chip calorimetry has been the different time sensitivity of XRD detectors and fast calorimeters. This has been solved by applying AC calorimetry in combination with time-resolved nanofocus X-ray diffraction measurements using a synchrotron beamline.^5,28^ AC calorimetry extends the applicable scanning rate to lower values to match the time resolution of the calorimeter and XRD detectors. Such combination allows for the detection of heat capacity changes but not the latent enthalpy during transformations. The development of FDSC chips with increased heat-flow resolution at relatively low scanning rates made the simultaneous acquisition of heat-flow curves and the associated WAXD patterns in a scanning-rate range of 20–200 K s^–1^ possible.^29^ Rosenthal et al. introduced an ultrafast chip-based nanocalorimeter to be used *in situ* with the high-intensity nanofocus X-ray beamline at ESRF^30^ to study the thermal behavior of polymer samples,^31,32^ self-assembled carbohydrate-functionalized gold nanoparticles,^33^ and high-energy materials.^34^

In this study, we combine FDSC with a fourth-generation high-energy synchrotron X-ray source to investigate the effect of a microfocused high-brilliance X-ray beam on an organic material, 4′-ethyl-4-(4-propyl-cyclohexyl)-biphenyl (BCH-52) (Figure 1). This is a liquid crystal compound with high thermal stability used for temperature calibration in DSC because of its defined transformation temperatures and high reversibility upon heating and cooling.^35^ The experimental setup is used to measure the energy absorption of this compound due to radiation and related temperature change. A beam damage of the sample can also be detected.

**Figure 1:**
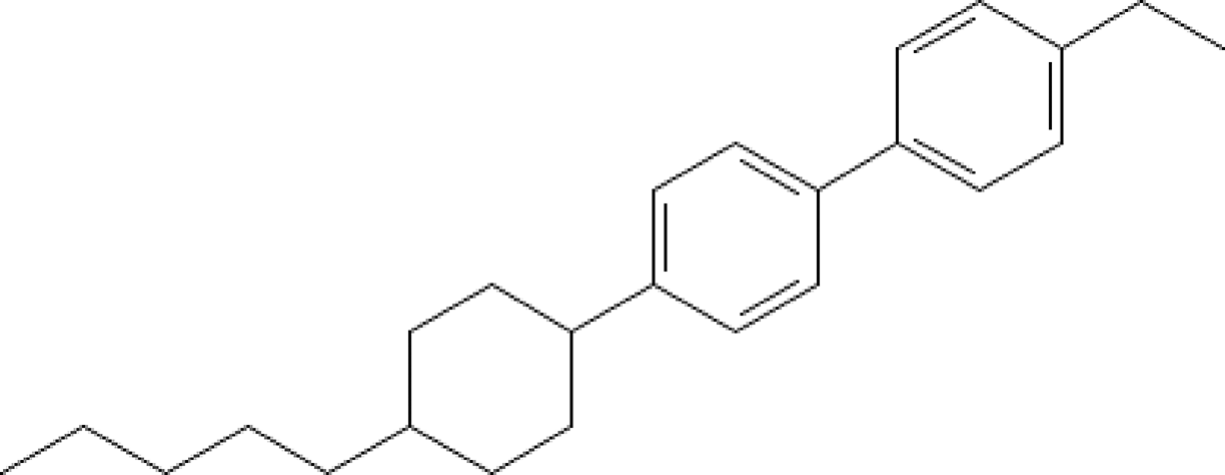
Chemical structure of 4′-ethyl-4-(4-propyl-cyclohexyl)-biphenyl (BCH-52).

Figure 2 displays FDSC heating curves of a BCH-52 sample with a mass of about 100 ng measured in the beamline without and with the X-ray beam. It illustrates the peak of the smectic B to nematic transition at about *T*_r_ = 150 °C and the nematic to isotropic liquid transition at about 167 °C. The heat-flow curve shifts exothermally because of the X-ray beam-energy absorption in the measurement system (sensor and sample). The measured heat flow, Φ, can be characterized by the sum of the sample response and the heat flow due to thermal losses, Φ_loss_. For a given ambient temperature of the sensor, Φ_loss_(*T*) is invariant with respect to the heating rate and hence to the power supplied to the calorimeter.^36^ Taking the additional contribution caused by the absorbed power from the beam, Φ_beam_, into account, the measured heat flow is
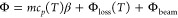
1where *m* is the sample mass, *c*_*p*_ is the specific heat capacity of the sample and β is the heating rate. Dynamic thermal equilibrium is quickly established, i.e., the power provided by the X-ray beam is compensated by heat losses into the surroundings.^5^ The difference of the heat flow for the heating curves recorded with and without beam gives the power supplied by the beam. The curves in Figure 2 lead to a beam contribution of . The absorption of an empty sensor is 0.051 mW. Thus, the resulting heat absorbed by the sample is .

**Figure 2:**
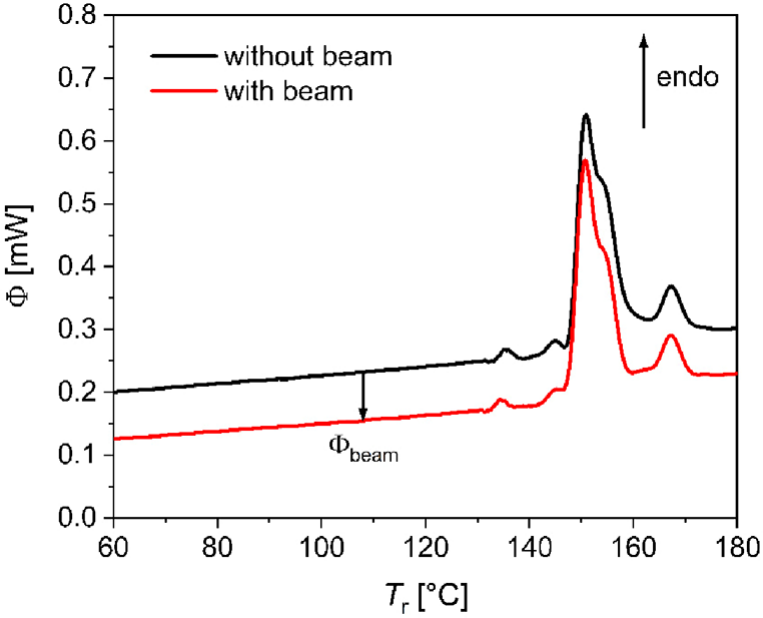
Effect of a 13 keV X-ray beam on the FDSC heating curve of BCH-52 measured at 1000 K s^–1^.

The experimentally determined heat absorbed by the sample is validated by a calculation of the absorbed power due to the interaction between photons and the sample. The photon flux φ is reduced exponentially when passing through matter:
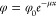
2where φ_0_ is the incident beam flux at the sample surface, *x* is the thickness of the sample, and μ is the linear attenuation coefficient. For the beam energy used in this study, μ is approximately equal to the photoelectric absorption coefficient^37^ and depends on the photon energy and material. We use here the value of the mass-energy absorption coefficient, μ_en_/ρ, which takes the kinetic energy lost as bremsstrahlung into account; this is a better estimation of the available energy associated with the incident radiation.^38^

The energy absorbed by the sample, φ_0_ – φ, is equal to the heat flow from the X-ray beam into the sample, Φ_beam_, and is a function of the beam flux, beam energy and sample material:
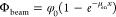
3

The energy of the X-ray beam, Φ_beam_, was calculated from the alignment data, and is equal to 0.521 mW in the 30 μm × 30 μm beam cross-section. Assuming for BCH-52 μ_en_/ρ = 2 cm^2^ g^–1^,^39^*x* = 10 μm, ρ = 0.94 g cm^–3^, and the aforementioned beam power, the total calculated absorbed energy by the sample is 0.03 mW. Energy absorption by Kapton windows, air, misalignment of the experimental setup, and misalignment of the sample on the sensor contribute to the uncertainty of this estimation. Considering the simplifications of the model and the uncertainties of the used material properties, this result agrees well with the measured value of 0.022 mW.

The beam filling mode during the measurements was 7/8 + 1, which leads to a modulation of the X-ray pulses and can affect the sample temperature.^40^ The complex irradiation pattern slightly increases the sample temperature during each pulse. However, due to the difference of several orders of magnitude between the irradiation pulse time (typically 20 ps) and the characteristic time of the calorimeter with the sample (ca. 1 ms), the heat capacity of the sample-calorimeter system acts as a low-pass filter that averages the heat pules of the X-ray source.

The temperature of the sensor membrane was controlled by the FDSC and the sample was coupled via an effective thermal resistance *R*_th_. If the sample is additionally heated by the X-ray beam, the temperature increase of the sample can be estimated by a simplified Fourier equation: Φ_sample_ = (1/*R*_th_)Δ*T*, where Φ_sample_ ≅ 0.022 mW is the heat absorption by the sample and Δ*T* is the temperature difference between the sample and sensor. The latter is a good approximation for the temperature increase of the sample. During melting of pure metals, the sensor temperature increases due to heating, while the sample temperature remains constant. Consequently, the low-temperature side of the melting peak has a constant slope of 1/*R*_th_. If we assume that the transformation peak at about 155 °C (Figure 2) behaves similarly, we can estimate 1/*R*_th_ to be approximately 0.14 mW K^–1^. This leads to Δ*T* ≈ 0.16 K and agrees well with the measured temperature difference of 0.2 K.

To quantify the beam damage, the sample was left in the X-ray beam at 25 °C for an extended period of time. FDSC heating curves were recorded after selected time intervals at a heating rate of 1000 K s^–1^, with presence of the beam. The damage of the sample caused by the X-ray beam is indicated by the change of the transformation peaks after exposure (Figure 3). Both peaks for the smectic B to nematic and the nematic to isotropic liquid transitions broaden and shift to lower temperatures. The curves recorded after beam exposure for 4–100 s show a continuous variation in peak shape with a decrease in temperature with increasing exposure time. No peak change occurred in similar measurements without the X-ray beam.

**Figure 3:**
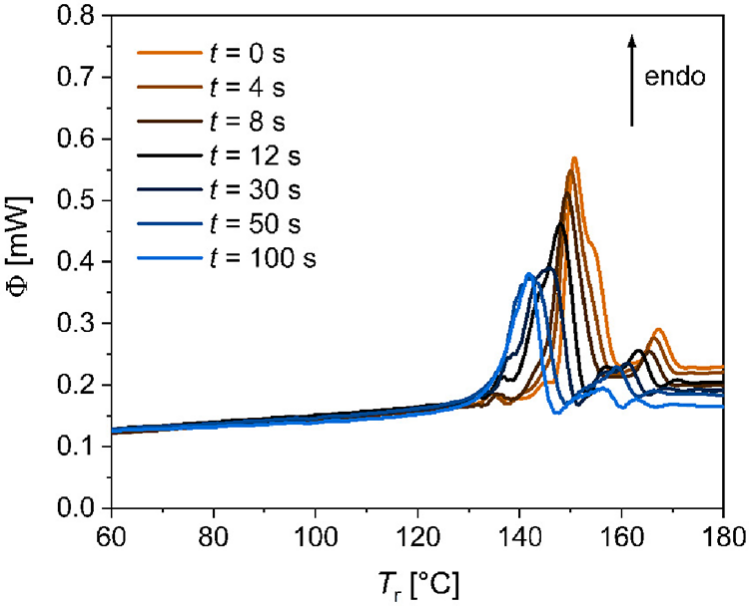
FDSC heating curves of BCH-52 at rates of 1000 K s^–1^ in the X-ray beam, revealing a significant effect of beam damage. Cooling between the heating cycles was performed at 1000 K s^–1^.

Structural changes were investigated using the simultaneously collected XRD patterns. First, the sample was exposed to synchrotron X-rays at room temperature and the XRD pattern was collected for 20 ms (Figure 4a, upper pattern). Subsequently, the sample was thermally cycled in the X-ray beam between 25 and 180 °C at rates of 1000 K s^–1^ (Figure 4b, upper image) to observe possible structural changes in the liquid state. Finally, an XRD pattern was collected at room temperature at conditions identical to those at the beginning of the experiment, after being irradiated by the X-ray beam for 200 s in total. A comparison of the XRD patterns at the beginning and end of the experiment is shown in Figure 4a. The background from the Si_3_N_*x*_ membrane of the sensor and the Kapton windows was not subtracted from the spectrum. A strong reflection occurred at *q* = 2.6958 Å^–1^ (not shown), which corresponds to a lattice spacing of 2.331 Å and results from the (111) lattice planes of a thin Al layer in the UFS sensor membrane. This peak also occurred in the XRD pattern of an empty sensor and was thus not considered for analysis. The sample peaks at 1.3567 Å^–1^ and 0.2832 Å^–1^ correspond to spacings of 4.631 and 22.186 Å, respectively. The smaller lattice spacing is assigned to the intermolecular parameter of BCH-52 and the larger one to the layer spacing of the smectic B phase. While the interlayer spacing is in good agreement with the value determined by Klämke and Haase,^41^ the intermolecular parameter is smaller than reported.^41^ After a total beam exposure time of 200 s, the peak of the layer spacing disappeared and the peak intensity corresponding to the intermolecular parameter decreased. No sample evaporation was observed; thus, the intensity decay results from sample degradation.

**Figure 4:**
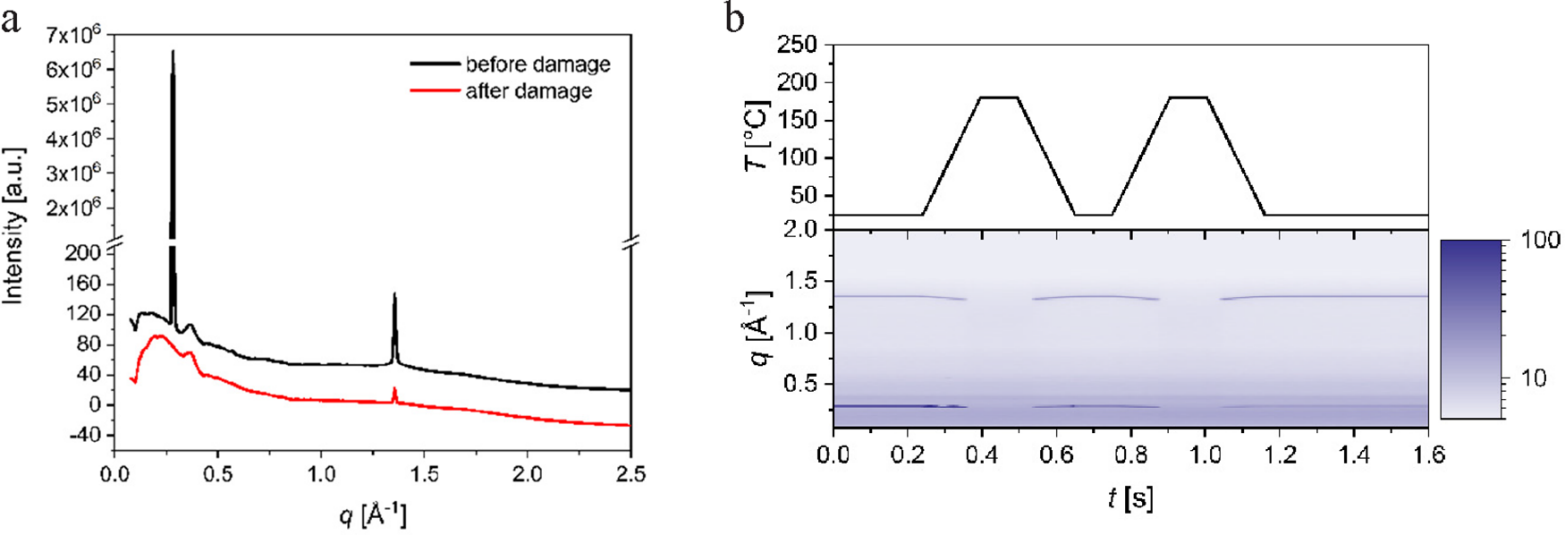
(a) X-ray diffraction (XRD) patterns collected at room temperature at the beginning and end of the experimental session after 200 s of beam exposure. (b) Time evolution of *in situ* XRD patterns (lower image) during thermal cycles between 25 and 180 °C at heating and cooling rates of 1000 K s^–1^ (upper image).

Figure 4b shows the time evolution of XRD patterns that were recorded every 1.4 ms *in situ* during a FDSC thermal cycle comprising a continuous sequence of heating and cooling segments, and the corresponding temperature program. Thermal expansion of the material is indicated by a slight shift of the peak at 1.3567 Å^–1^ to lower *q* values. However, the interlayer spacing of BCH-52 is temperature independent. After beam exposure in the isotropic liquid, the intensity of this peak decreases rapidly by several orders of magnitude, whereas the intensity of the peak at higher *q* decays slowly. Therefore, the intermolecular spacing is less affected by the beam damage than the interlayer spacing. The first indication of beam damage occurs after the first heating and cooling cycle, i.e., after about 0.1 s beam exposure in the isotropic liquid state. The X-ray diffraction technique employed in this experiment did not provide information on modifications of the molecules, and thus does not allow to describe the exact nature of sample damage. From the literature, it is known that several processes are involved in the radiation damage of organic molecules, such as formation of hydrogen^42^ and other gases,^43^ which leads to radicalization of the compound.^44,45^ The higher molecular stability at lower temperatures^42^ indicates the important role of molecular mobility in radiation damage. We assume that in the crystalline and liquid crystalline (i.e., smectic B and nematic) states, the reduced molecular mobility leads to a higher probability of radical recombination. This generates less structural changes than in the isotropic liquid, where molecular mobility is high. Therefore, the sample is more prone to beam damage when irradiated in the isotropic liquid compared to the crystalline or liquid crystalline states.

To confirm the lack of thermal degradation of the sample due to a potentially absorbed thermal energy from the X-ray beam, a stability estimation measurement was performed *ex situ* via FDSC.^46^ The experimental details and results are given in the Supporting Information. The measurements reveal that the sample is resistant to thermal damage up to 320 °C in thermal pulses of 5 ms, where only sample evaporation is observed. The synchrotron X-ray pulses are 9 orders of magnitude shorter and thus the thermal stability of the compound is expected to be higher at such short time scales. As no sample evaporation was observed during the *in situ* synchrotron experiments, the temperature increase due to the X-ray pulses must be lower than the exposure temperature in the stability estimation experiment. We thus conclude that the intensity decay in the XRD pattern can only result from a sample degradation due to radiation damage.

In conclusion, *in situ* combination of a high-brilliance X-ray source with FDSC enables the simultaneous determination of structural changes and thermal properties. It also allows to determine the influence of the X-ray beam on the sample. The absorbed beam flux is measured by the additional contribution of the measured heat flow, and the temperature change by the shift of the phase transformation temperature compared to measurements of a nonirradiated sample. Irreversible beam damage is detected by a temperature shift and shape change of the transformation peaks in the heat-flow curves and by changes in the diffraction patterns.

For the setup used we found for the organic compound BCH-52 a small change of the average temperature by 0.2 K due to the temperature control of the DSC, an absorbed energy of 0.022 mW (approximately 220 W g^–1^), and a significant beam damage after 0.55 s total exposure time including ca. 0.1 s at 180 °C in the isotropic liquid state. The absorbed energy is small compared to the heat-flow signal of the smectic B to nematic phase transformation in BCH-52. This indicates that the sample is not damaged by the thermal effect of the X-ray beam, but by radiation damage that generates structural changes in the sample. This has also been confirmed by thermal stability measurements described in the Supporting Information. The interlayer spacing of the smectic B phase is affected more than the intermolecular parameter of the compound, and the ordering of the liquid phase decreases upon beam exposure.

The effect of a beam on materials analysis depends on the nature of the sample and on beam type and energy.^9^ Materials with high absorption coefficients suffer from stronger beam heating, and similar effects have also been observed in a scanning electron microscope. The *in situ* Flash DSC technique described in this work measures the average energy absorbed by the beam-sample interaction, while the diffraction data reveals changes in the ordering of the phase. The changes in the transformation behavior measured in the heat-flow curves are very sensitive but not specific toward the molecular level. Such information may be obtained by nondestructive *ex situ* spectroscopic sample analysis prior to and after the experiment.

The fourth-generation high-energy synchrotron increases performance by a factor 100, but its high brilliance has a negative effect on sample stability in the beam. Due to the high heating and cooling rates, FDSC measurements take only several milliseconds, which is fast enough to perform thermal characterization and avoid sample damage. Thus, we demonstrate the suitability of FDSC as one of the possible ways to overcome the problem of sample stability within an X-ray beam. The agreement between the time constant of the FDSC measurements and the acquisition time of X-ray detectors renders true *in situ* measurements possible.

## Experimental Methods

The material investigated was 4′-ethyl-4-(4-propyl-cyclohexyl)-biphenyl (BCH-52) purchased from Merck KGaA. This material shows three phase transformations:^1^ crystalline to smectic B at around 40 °C,^2^ smectic B to nematic at about 146.8 °C, and^3^ nematic to isotropic liquid at about 164.8 °C.^35^

The diffraction experiments were performed using a monochromatic high-brilliance X-ray beam at the ID 13 beamline at the European Synchrotron Radiation Facility (ESRF, Grenoble, France) with an energy of 13 keV (wavelength of 0.09537 nm) and a photon flux of 2.5 × 10^11^ s^–1^ (recorded during the beam-alignment procedure), providing a beam power of 0.521 mW in the 30 μm × 30 μm beam cross-section. A Dectris EigerX 4 M detector with a resolution of 2070 × 2167 pixels and 75 μm pixel size was positioned at 109 mm from the sample.

The synchrotron operated in the 7/8 + 1 filling mode with a refill frequency of 1 h. In this mode, 868 bunches are equally spread throughout 7/8 of the 843 m long storage ring and a single bunch is located in the remaining 1/8 of the ring. Thus, each bunch circulates in the ring with a frequency of about 355 kHz and the frequency of the pulses interacting with the sample is on the order of MHz, which is 3 orders of magnitude higher than the sampling frequency of the X-ray detector and the Flash DSC. The typical pulse duration is 20 ps. Therefore, the effect of the time pulses of the synchrotron radiation was negligible in the experiment and we assumed the X-ray beam to be continuous. All measurements were performed between the storage-ring refill events to avoid fluctuations of the X-ray beam intensity.

A Flash DSC 2+ (Mettler-Toledo, Switzerland) instrument with a UFS 1 sensor was utilized. The active zone of this sensor contains a thin Al layer, which generates characteristic reflections that can be easily eliminated from the diffraction patterns. The sensor was purged with Ar at a flow rate of about 40 mL min^–1^. Details of the instrument and sample handling are presented in refs (12, 15).

For the purpose of the *in situ* FDSC measurements, the Flash DSC 2+ was equipped with an external sensor support, which was placed vertically in the beam path (for details see the Supporting Information). The X-ray beam only illuminated the sample site of the FDSC sensor.

## Supporting Information Available

Details of the external sensor support of a Flash DSC 2+ device, its modification for *in situ* synchrotron diffraction and of the sample thermal stability (PDF)
Transparent Peer Review report available (PDF)
