# Supplementary material for: Energy Absorption and Beam Damage during Microfocus Synchrotron X-ray Diffraction
Source: J Phys Chem Lett. 2024 Jun 7;15(24):6286–91. doi: 10.1021/acs.jpclett.4c00497 (PMC11194812; doi:10.1021/acs.jpclett.4c00497)
Supplement: Supplementary file 1 — jz4c00497_si_001.pdf [file jz4c00497_si_001.pdf]

Supporting information

Energy Absorption and Beam Damage During Micro-Focus Synchrotron X-Ray Diffraction

Štefan T. Stanko[a], Jürgen E. K. Schawe[a,b], Florian Spieckermann[c], Jürgen Eckert[c,d], Jörg F. Löffler[a]

[a] Laboratory of Metal Physics and Technology, Department of Materials, ETH Zurich, 8093 Zurich, Switzerland

[b] Mettler-Toledo GmbH, Analytical, 8606 Nänikon, Switzerland.

[c] Department of Materials Science, Chair of Materials Physics, Montanuniversität Leoben, 8700 Leoben, Austria

[d] Erich Schmid Institute of Materials Science, Austrian Academy of Sciences, 8700 Leoben, Austria

EXTERNAL SENSOR SUPPORT

For the purpose of the *in situ* FDSC measurements, a Flash DSC 2+ (Mettler-Toledo, Switzerland) was equipped with an external sensor support, which was placed vertically in the beam path. An opening was drilled at the bottom side of the sensor to allow the X-ray beam to reach the sample. Both sides were closed by a polyimide window and the furnace was purged with Ar to prevent sample oxidation. All measurements were performed between 25 °C and 180 °C at heating and cooling rates of 1000 K s^{-1}. The X-ray beam only illuminated the sample site of the FDSC sensor (Figure S1).

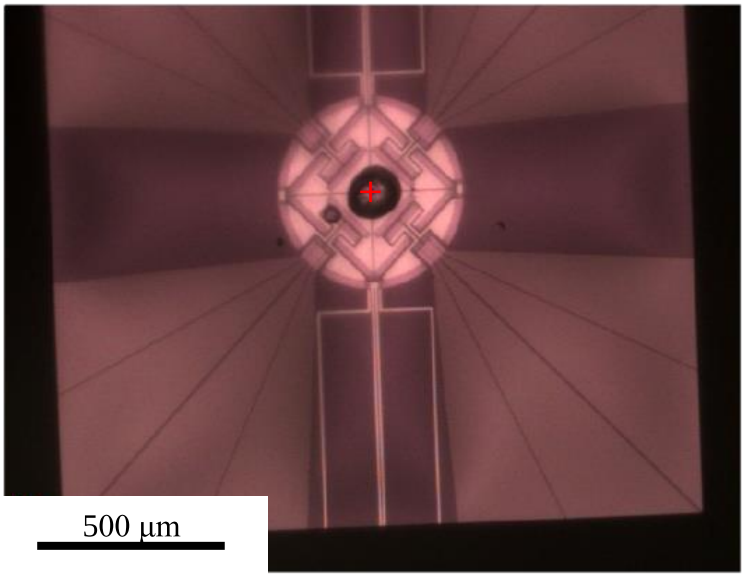

Figure S1. FDSC UFS sensor with a BCH-52 sample. The red cross indicates the beam position and size.

In order to include the FDSC setup in the beamline ID13 at ESRF in Grenoble, a break-out box was built using the sensor-holder and pin-cables spare parts of a Flash DSC 2+ device (see Fig. S2). This box contains the sensor-support holder (Fig. S2(b)), as well as the Flash DSC 2+ spare-parts sensor holder (Mettler-Toledo part No (MT) 30474853) and pin cable (MT 30472386) (Figure S2(c)). These parts were mounted on a dedicated support for the ID 13 sample stage (Figure S2(c)). Such a design allows for maximum compatibility with the Flash DSC device, as no new connectors are needed. Triggering of the ID13 beamline and detector was achieved using the USB trigger extension of the Flash DSC device.

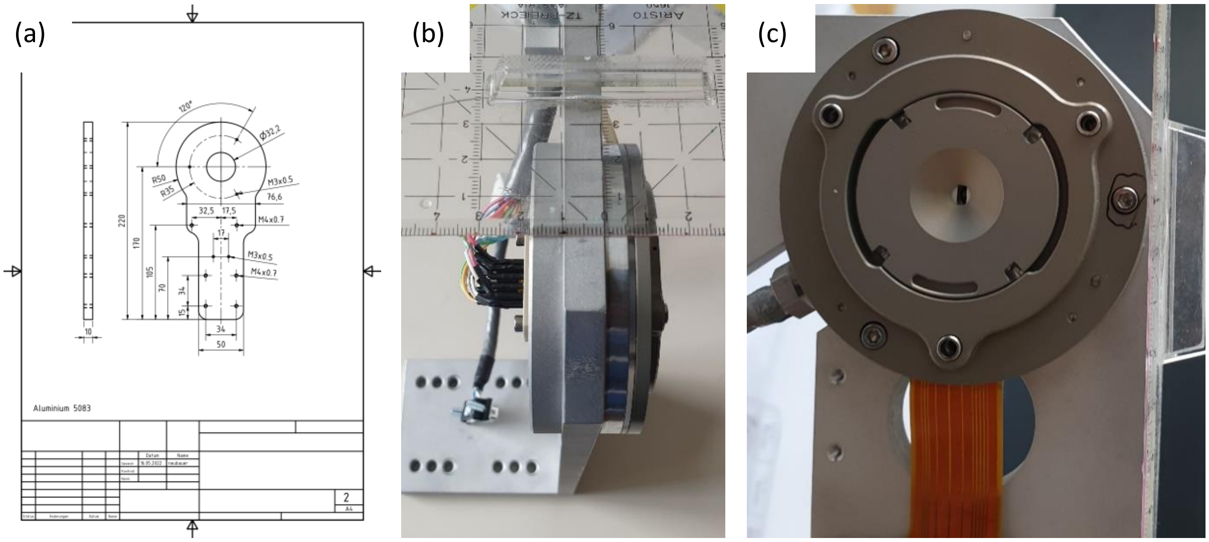

Figure S2. Representation of the Mettler-Toledo sensor holder adapted for *in situ* synchrotron diffraction. (a) Schematic drawing of the sensor-support holder. (b) Side view of the holder. (c) Front view of the holder. Control is provided by the Flash DSC device, which allows to fully record the calorimetric signal during the experiment, including effects caused by the synchrotron X-ray beam.

THERMAL STABILITY OF BCH-52

In order to evaluate the thermal stability of BCH-52 by potential heating due to absorption of the picosecond synchrotron X-ray pulses, we deployed a calorimetric technique originally developed to study the short-time stability of organic materials (1). For such studies, the Flash DSC 2+ with a UFH 1 sensor was used and purged with Ar at a flow rate of 40 ml min^{-1}. As shown in Figure S3(a), the temperature program consists of a sequence of analysis steps between 25 °C and 180 °C, equivalent to the *in situ* XRD experiments performed, plus thermal stress exposure steps between 180 °C and an elevated temperature T_i, where T_i is sequentially increased up to 320 °C

in 10 °C steps. The thermal stress exposure steps were performed with heating and cooling ramps of 10,000 K s^{-1} and isothermal steps of Δt = 5 ms in between.

The heat flow curves of the sample are shown in the inset to Fig. S3(b) and were recorded during heating up to 180 °C at a rate of 1000 K s^{-1} (analysis step) after the various thermal stress exposure steps. As can be seen in Fig. S3(b), the temperature and shape of the transformation peaks remained unchanged, and only the peak area and heat flow of the baseline decreased. This indicates a mass loss due to some evaporation in the liquid state, but no further damage to the sample.

For quantitative analysis, we defined a stability parameter, $\Sigma_{H/C}$, obtained by dividing the enthalpy of the transformation peak at 155 °C by the heat capacity of the sample before the transformation. Figure S3(b) shows $\Sigma_{H/C}$ as a function of exposure temperature T_i, and illustrates that the stability parameter does not change with increasing T_i. In fact, the intensity of the transformation peak and the heat capacity decrease with continuous sample evaporation, but no thermal degradation is observed, even when overheating to 140 K above the maximum temperature of the *in situ* synchrotron X-ray experiment. This illustrates that thermal decomposition of the sample by its potential heating due to absorption of the picosecond X-ray pulses is unlikely.

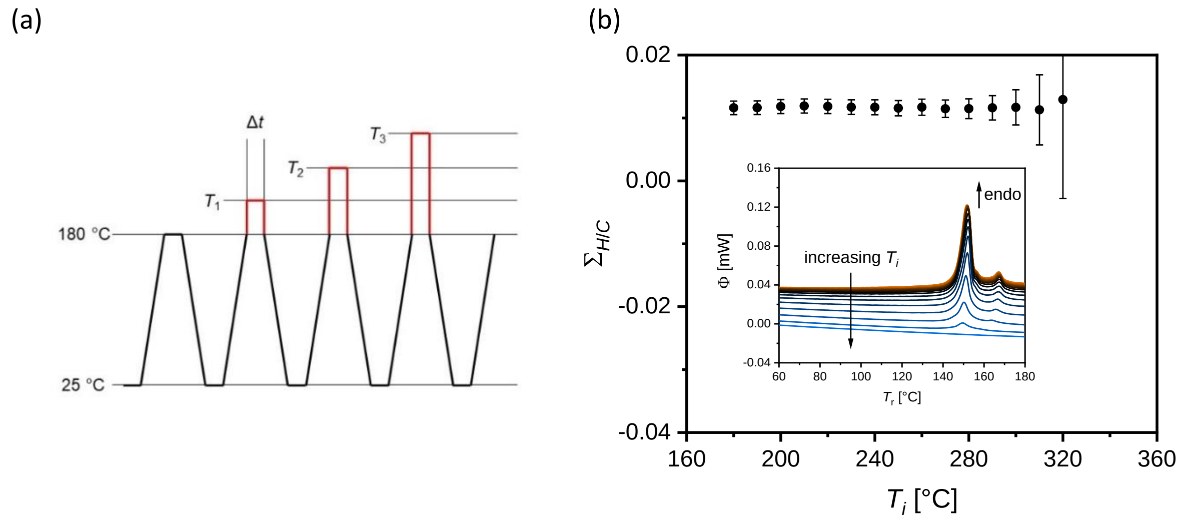

Figure S3. (a) Temperature program consisting of a sequence of analysis steps between 25 °C and 180 °C, plus thermal stress exposure steps between 180 °C and T_i, where T_i is sequentially increased up to 320 °C in 10 °C steps. (b) Heating curves of BCH-52 (inset) measured at a rate of 1000 K s^{-1}, and stability parameter, $\Sigma_{H/C}$, as a function of overheating temperature T_i. The error bars were determined from the uncertainties of measured heat capacity before the transformation at around 155 °C.

REFERENCES

(1) Schawe, J. E. K.; Ziegelmeier, S. Determination of the thermal short time stability of polymers by fast scanning calorimetry. *Thermochim. Acta*, **2016**, *623*, 80–85
