# Supplementary material for: Energy Absorption and Beam Damage during Microfocus Synchrotron X-ray Diffraction
Source: J Phys Chem Lett. 2024 Jun 7;15(24):6286–91. doi: 10.1021/acs.jpclett.4c00497 (PMC11194812; doi:10.1021/acs.jpclett.4c00497)
Supplement: Supplementary file 2 — jz4c00497_si_002.pdf [file jz4c00497_si_002.pdf]

jz-2024-00497n.R1

Name: Peer Review Information for "Energy Absorption and Beam Damage During Micro-Focus Synchrotron X-ray Diffraction"

First Round of Reviewer Comments

Reviewer: 1

Comments to the Author

The paper fits into a consolidated tradition of the ESRF ID13 beamline in combining differential scanning calorimetry and XRD/WAXS focusing in this case on radiation damage aspects. The problem is interesting and particularly relevant for scientists working with X-ray micro-/nano-beams. However, the current quality of the discussion is insufficient to provide significant new physico-chemical insights which justify the publication on The Journal of Physical Chemistry Letters. It might be reconsidered if the following issues are properly addressed:

1) The paper completely overlooks the time structure of the synchrotron radiation, which is pulsed in nature, giving rise to instantaneous energy absorption by the sample which are not constant in time and are not described by the simple models adopted in the article. In this context, the details about the storage ring filling mode should be also reported.

2) Connected to the previous point the statement reported on page 10 “the sample is not damaged by the thermal effect of the X-ray beam” should be supported by more refined simulations which provide the variation of the sample temperature as a function of time covering also timescales comparable to the X-ray pulse duration (see e.g. Rev. Mod. Phys. 90 (2018) 025007; J. Synchrot. Radiat. 27 (2020) 1662-1673).

3) The possible physico-chemical mechanisms responsible for the observed beam damage should be better discussed (e.g. effect of secondary electrons, etc.).

4) The authors performed their study on a standard compound commonly used for temperature calibration in DSC, but, since this investigation has not a special application interest, but can be interesting from a fundamental perspective, they should try to generalize their conclusions to

provide more general physico-chemical insights which could be adopted by the scientists working with X-ray micro-/nano-beams to predict and rationalize beam damage effects.

Reviewer: 2

Comments to the Author

Although it is know that high intensity x-ray beams can damage materials, the novelty here lays in the use of the calorimetric signal do estimate the energy input. The results are not spectacular but worth publication.

The authors may take the following minor remarks into account:

1. In relation to equation (3): The beam power (incident beam flux) has not been mentioned before as it is only written in the experimental section at the end of the manuscript. Simply add this value here. It should also be mentioned how this value is obtained: measured or calculated? If measured; where did that take place? Information is missing.

2. A peak shift of 0.2 K was observed when the beam is on: It is written that this is an expected shift. Can this expected shift be calculated?

Author's Response to Peer Review Comments:

Reviewer(s)' Comments to Author:

Reviewer: 1

Recommendation: This paper may be publishable, but major revision is needed; I would like to be invited to review any future revision.

Comments:
The paper fits into a consolidated tradition of the ESRF ID13 beamline in combining differential scanning calorimetry and XRD/WAXS focusing in this case on radiation damage aspects. The problem is interesting and particularly relevant for scientists working with X-ray micro-/nano-beams. However, the current quality of the discussion is insufficient to provide significant new physicochemical insights which justify the publication on The Journal of Physical Chemistry Letters. It might be reconsidered if the following issues are properly addressed:

1) The paper completely overlooks the time structure of the synchrotron radiation, which is pulsed in nature, giving rise to instantaneous energy absorption by the sample which are not constant in time and are not described by the simple models adopted in the article. In this context, the details about the storage ring filling mode should be also reported.

2) Connected to the previous point the statement reported on page 10 “the sample is not damaged by the thermal effect of the X-ray beam” should be supported by more refined simulations which provide the variation of the sample temperature as a function of time covering also timescales comparable to the X-ray pulse duration (see e.g. Rev. Mod. Phys. 90 (2018) 025007; J. Synchrot. Radiat. 27 (2020) 1662-1673).
3) The possible physico-chemical mechanisms responsible for the observed beam damage should be better discussed (e.g. effect of secondary electrons, etc.).
4) The authors performed their study on a standard compound commonly used for temperature calibration in DSC, but, since this investigation has not a special application interest, but can be interesting from a fundamental perspective, they should try to generalize their conclusions to provide more general physico-chemical insights which could be adopted by the scientists working with X-ray micro-/nano-beams to predict and rationalize beam damage effects.

Additional Questions:

Urgency: Moderate

Significance: High

Novelty: High

Scholarly Presentation: Moderate

Is the paper likely to interest a substantial number of physical chemists, not just specialists working in the authors' area of research?: Yes

Reviewer: 2

Recommendation: This paper is publishable subject to minor revisions noted. Further review is not needed.

Comments:
Although it is know that high intensity x-ray beams can damage materials, the novelty here lays in the use of the calorimetric signal do estimate the energy input. The results are not spectacular but worth publication.

The authors may take the following minor remarks into account:

1) In relation to equation (3): The beam power (incident beam flux) has not been mentioned before as it is only written in the experimental section at the end of the manuscript. Simply add this value here. It should also be mentioned how this value is obtained: measured or calculated? If measured; where did that take place? Information is missing.
2) A peak shift of 0.2 K was observed when the beam is on: It is written that this is an expected shift. Can this expected shift be calculated?

Additional Questions:

Urgency: High

Significance: High

Novelty: Moderate

Scholarly Presentation: High

Is the paper likely to interest a substantial number of physical chemists, not just specialists working in the authors' area of research?: Yes

Editor’s Non-Scientific Changes to Author:

1) Please submit your publication files without any markups. Please include annotated version(s) of your revised publication file(s) with colored text or highlights indicating the revisions that you have made as "Supporting Information for Review Only."
2) Title: In both the main manuscript file and the Supporting Information, set the title in title case, with the first letter of each principal word capitalized.
3) References: In both the main file and the supporting information, fix the style of all references to use JPCL formatting (check all references carefully). ***JPC Letters reference formatting requires that journal references should contain: () around numbers; author names; article title (titles entirely in title case or entirely in lower case); abbreviated journal title (italicized); year (bolded); volume (italicized); and pages (first-last). Book references should contain author names; book title (in the same pattern); publisher; city; and year. Websites must include date of access.
4) References: URLs are not preferred references because website content can be modified and, consequently, the reference information may lack permanence.
5) TOC Graphic: Please resize the TOC graphic per journal guidelines (2 in x 2 in) and move to the correct position (on the same page as the abstract).

Supporting Information: Please number SI pages in the following format: “S1, S2…”

6) Supporting Information: Please number SI pages in the following format: “S1, S2…”

Štefan Stanko
ETH Zurich
Vladimir-Prelog-Weg 1-5/10 Zurich,
CH 8093 stefan.stanko@mat.ethz.ch
+41 44 633 64 94

[27th March, 2024]

We would like to express our appreciation to the editor and all reviewers for their valuable comments on our manuscript, and the opportunity to improve the paper. We are pleased to hear

that the reviewers found our manuscript interesting and relevant for scientists working in the X-ray beam community and physical chemistry in general, and that it is therefore worth publishing in the *Journal of Physical Chemistry Letters*. Revisions in the manuscript are highlighted in yellow.

In the following we would like to reply to the reviewers' comments point by point:

(1) **Reviewer 1**: *The paper completely overlooks the time structure of the synchrotron radiation, which is pulsed in nature, giving rise to instantaneous energy absorption by the sample which are not constant in time and are not described by the simple models adopted in the article. In this context, the details about the storage ring filling mode should be also reported.*

Thank you for raising this concern. We report the details about the beam filling mode during the experiment to the manuscript. However, the time structure of the synchrotron radiation is several orders of magnitude faster than the time constants of the measuring equipment used and we are not able to resolve it during the performed experiments.

The following paragraph is are added:

The beam filling mode during the measurements was 7/8+1, which leads to a modulation of the X-ray pulses and can affect the sample temperature (40). The complex irradiation pattern slightly increases the sample temperature during each pulse. However, due to the difference of several orders of magnitude between the irradiation pulse time (typically 20 ps) and the characteristic time of the calorimeter with the sample (ca. 1 ms), the heat capacity of the sample-calorimeter system acts as low-pass filter that averages the heat pules of the X-ray source.

In the experimental section we added:

The synchrotron operated in the 7/8 + 1 filling mode with a refill frequency of 1 hour. In this mode, 868 bunches are equally spread throughout 7/8 of the 843 m long storage ring and a single bunch is located in the remaining 1/8 of the ring. Thus, each bunch circulates in the ring with a frequency of about 355 kHz and the frequency of the pulses interacting with the sample is on the order of MHz, which is three orders of magnitude higher than the sampling frequency of the X-ray detector and the Flash DSC. The typical pulse duration is 20 ps. Therefore, the effect of the time pulses of the synchrotron radiation was negligible in the experiment and we assumed the X-ray beam to be continuous. All measurements were performed between the storage-ring refill events to avoid fluctuations of the X-ray beam intensity.

(2) **Reviewer 1**: *Connected to the previous point the statement reported on page 10 "the sample is not damaged by the thermal effect of the X-ray beam" should be supported by more refined simulations which provide the variation of the sample temperature as a function of time*

covering also timescales comparable to the X-ray pulse duration (see e.g. Rev. Mod. Phys. 90 (2018) 025007; J. Synchrot. Radiat. 27 (2020) 1662-1673).

Thank you for this suggestion. We are aware of the temperature variations of the sample due to the Xray pulses. The absorbed power due to irradiation averaged over 1 ms is about 22 μW. This value is measured directly and leads to a temperature increase of 0.2 K. The power required for the main

transformation measured is about 20 times higher. This low thermal impact of the radiation beam on the sample leads us to conclude that this cannot be the reason for the rapid decomposition during irradiation. Thank you also for the suggestion of model calculations, but we think that this would overload the letter and may be performed in the context of subsequent publications. The references are very helpful and have been added to the manuscript.

(3) **Reviewer 1**: *The possible physico-chemical mechanisms responsible for the observed beam damage should be better discussed (e.g. effect of secondary electrons, etc.).*

Thank you for this comment. The detailed characterization of the mechanisms of beam damage can be revealed by further spectroscopy experiments of the analyzed samples, however, this is not the topic of this work. The measuring techniques used in this paper do not allow to elucidate the particular mechanisms of beam damage in BCH-52: the changes in the transformation behavior measured in the heat flow curves are sensitive but not specific, and the X-ray diffraction technique used in this work does not provide information on molecule modifications.

For clarification, we have added the following sections to the manuscript:

The X-ray diffraction technique employed in this experiment did not provide information on
modifications of the molecules, and thus does not allow to describe the exact nature of the sample
damage.

The *in situ* Flash DSC technique described in this work measures the averaged energy absorbed by the beam-sample interaction, while the diffraction data reveals changes in the ordering of the phase. The changes in the transformation behavior measured in the heat-flow curves are very sensitive but not specific towards the molecular level. Such information may be obtained by non-destructive *ex situ* spectroscopic sample analysis prior to and after the experiment.

(4) **Reviewer 1**: *The authors performed their study on a standard compound commonly used for temperature calibration in DSC, but, since this investigation has not a special application interest, but can be interesting from a fundamental perspective, they should try to generalize their conclusions to provide more general physico-chemical insights which could be adopted by the scientists working with X-ray micro-/nano-beams to predict and rationalize beam damage effects.*

Thank you for the comment which relates to the previous point. Our main intension was to introduce and present a relatively simple technique to measure absorbed energy, sample heating and beam damage. We concluded that one of the possible ways to prevent beam damage is to limit the duration of the beam exposure and thus the time of the experiments to a minimum. Flash DSC allows precise measurements on a short time scale and the fourth-generation high-energy synchrotron still provides sufficient diffraction pattern intensity for sample characterization. We

selected a substance forming liquid crystalline phases with defined transformations and high thermal stability, and assumed a reduced stability with respect to irradiation in the liquid state.

(5) **Reviewer 2**: *In relation to equation (3): The beam power (incident beam flux) has not been mentioned before as it is only written in the experimental section at the end of the manuscript. Simply add this value here. It should also be mentioned how this value is obtained: measured or calculated? If measured; where did that take place? Information is missing.*

Thank you for pointing this out. The beam power is now mentioned earlier in the manuscript. It was calculated from the photon count, which was recorded during the beam-alignment procedure using a diode measuring the beam intensity.

The following changes in the manuscript have been made:

The energy of the X-ray beam, Φ_{beam}, was calculated from the alignment data, and is equal to

0.521 mW in the 30 μm × 30 μm beam cross-section.

The diffraction experiments were performed using a monochromatic high-brilliance X-ray beam at the ID 13 beamline at the European Synchrotron Radiation Facility (ESRF, Grenoble, France) with an energy of 13 keV (wavelength of 0.09537 nm) and a photon flux of 2.5×10^{11} s^{-1} (recorded during the beam-alignment procedure), providing a beam power of 0.521 mW in the 30 μm × 30 μm beam crosssection.

(6) **Reviewer 2**: *A peak shift of 0.2 K was observed when the beam is on: It is written that this is an expected shift. Can this expected shift be calculated?*

Thank you for this remark. We are indeed able to predict the temperature shift based on the slope of the transformation peak in the heating curves. This is equivalent to the effective thermal resistance. The corresponding calculation has been added to the manuscript:

The temperature of the sensor membrane was controlled by the FDSC and the sample was coupled via an effective thermal resistance R_{th}. If the sample is additionally heated by the X-ray beam, the temperature increase of the sample can be estimated by a simplified Fourier equation: $\Phi_{sample} = (1/R_{th})\ \Delta T$, where $\Phi_{sample} \cong 0.022$ mW is the heat absorption by the sample and ΔT is the temperature difference between the sample and sensor. The latter is a good approximation for the temperature increase of the sample. During melting of pure metals, the sensor temperature increases due to heating, while the sample temperature remains constant. Consequently, the low-temperature side of the melting peak has a constant slope of $1/R_{th}$. If we assume that the transformation peak at about 155 °C (Figure 2) behaves similarly, we can estimate $1/R_{th}$ to be approximately 0.14 mW K^{-1}. This leads to $\Delta T \approx 0.16$ K and agrees well with the measured temperature difference of 0.2 K.

Thank you for your consideration.

Yours sincerely, on behalf of all authors,

Štefan Stanko
Department of Materials
ETH Zurich

jz-2024-00497n.R2

Name: Peer Review Information for "Energy Absorption and Beam Damage During Micro-Focus Synchrotron X-ray Diffraction"

Second Round of Reviewer Comments

Reviewer: 2

Comments to the Author

earlier comments have been properly accounted for!

Reviewer: 1

Comments to the Author

The authors have responded to my objections only partially. In particular, in the experimental section they state that “the effect of the time pulses of the synchrotron radiation was negligible in the experiment and we assumed the X-ray beam to be continuous”. This simplification may be valid to discuss the calorimetric data, but it does not guarantee that transient temperature increases in the picosecond timescale (i.e. the timescale of the typical X-ray pulse duration) are not affecting the sample structure. The authors report that “The absorbed power due to irradiation averaged over 1 ms is about 22 μW”, however this is only the average power on a timescale that is order of magnitudes longer than the timescale of the typical X-ray pulse duration.

Related to this issue, if the authors conclude that the radiation damage has not a thermal origin, they must discuss the possible physico-chemical mechanisms responsible for the observed beam damage, but no explanation has been added in the revised version. If XRD and calorimetry are not suitable to provide sufficient insights, they should combine other experimental/modelling techniques.

Given the considerations mentioned above and considering that, as stated also by Reviewer 2, “the results are not spectacular”, I think that a better discussion of the physico-chemical mechanisms connected to the role of beam heating and to the origin of the observed radiation damage are mandatory to justify the publication on The Journal of Physical Chemistry Letters.

Author's Response to Peer Review Comments:

Reviewer(s)' Comments to Author:

Reviewer: 2

Recommendation: This paper represents a significant new contribution and should be published as is.

Comments:

earlier comments have been properly accounted for!

Additional Questions:

Urgency: High

Significance: High

Novelty: Moderate

Scholarly Presentation: High

Is the paper likely to interest a substantial number of physical chemists, not just specialists working in the authors' area of research?: Yes

Reviewer: 1

Recommendation: This paper is probably publishable, but major revision is needed; I do not need to see future revisions.

Comments:

The authors have responded to my objections only partially. In particular, in the experimental section they state that "the effect of the time pulses of the synchrotron radiation was negligible in the experiment and we assumed the X-ray beam to be continuous". This simplification may be valid to discuss the calorimetric data, but it does not guarantee that transient temperature increases in the picosecond timescale (i.e. the timescale of the typical X-ray pulse duration) are not affecting the sample structure. The authors report that "The absorbed power due to irradiation averaged over 1 ms is about 22 μW", however this is only the average power on a timescale that is order of magnitudes longer than the timescale of the typical X-ray pulse duration.

Related to this issue, if the authors conclude that the radiation damage has not a thermal origin, they must discuss the possible physico-chemical mechanisms responsible for the observed beam damage, but no explanation has been added in the revised version. If XRD and calorimetry are not suitable to provide sufficient insights, they should combine other experimental/modelling techniques.

Given the considerations mentioned above and considering that, as stated also by Reviewer 2, "the results are not spectacular", I think that a better discussion of the physico-chemical mechanisms connected to the role of beam heating and to the origin of the observed radiation damage are mandatory to justify the publication on The Journal of Physical Chemistry Letters.

Additional Questions:

Urgency: Moderate

Significance: High

Novelty: Moderate

Scholarly Presentation: High

Is the paper likely to interest a substantial number of physical chemists, not just specialists working in the authors' area of research?: Yes

Štefan Stanko
ETH Zurich
Vladimir-Prelog-Weg 1-5/10
Zurich, CH 8093
stefan.stanko@mat.ethz.ch
+41 44 633 64 94

[25th May, 2024]

We want to thank the editor and reviewers for their insightful comments and for giving us the chance to improve the paper. We hope that the changes made to the manuscript have addressed all points of concern. Revisions in the manuscript are highlighted in yellow.

In the following we would like to reply to the reviewers' comments:

> **Reviewer 1**: *The authors have responded to my objections only partially. In particular, in the experimental section they state that "the effect of the time pulses of the synchrotron radiation was negligible in the experiment and we assumed the X-ray beam to be continuous". This simplification may be valid to discuss the calorimetric data, but it does not guarantee that transient temperature increases in the picosecond timescale (i.e. the timescale of the typical X-ray pulse duration) are not affecting the sample structure. The authors report that "The absorbed power due to irradiation averaged over 1 ms is about 22 µW", however this is only the average power on a timescale that is order of magnitudes longer than the timescale of the typical X-ray pulse duration.*
>
> *Related to this issue, if the authors conclude that the radiation damage has not a thermal origin, they must discuss the possible physico-chemical mechanisms responsible for the observed beam damage, but no explanation has been added in the revised version. If XRD and calorimetry are not suitable to provide sufficient insights, they should combine other experimental/modelling techniques.*
>
> *Given the considerations mentioned above and considering that, as stated also by Reviewer 2, "the results are not spectacular", I think that a better discussion of the physico-chemical mechanisms connected to the role of beam heating and to the origin of the observed radiation damage are mandatory to justify the publication on The Journal of Physical Chemistry Letters.*

Thank you for pointing this out. We performed additional experiments to assess the thermal stability of the sample. During this experiment we showed that the sample is thermally stable up to 320 °C in thermal pulses of 5 ms. Evaporation of the sample was the only observed phenomenon.

The X-ray pulses of the synchrotron are several orders of magnitude shorter (typically 20 ps) and the expected temperature increase due to X-ray absorption is less than the 140 K achieved during the thermal stability experiment. For shorter pulses, we expect higher thermal stability of the material.

During the *in situ* synchrotron experiment, no sample evaporation was observed. Thus, we assume that the temperature increase caused by synchrotron pulses is even lower than during the thermal stability measurement, and it would not cause thermal decomposition of the sample.

The following sentence was added in the abstract:

The aim of this paper is to investigate the influence of radiation on sample behavior.

The following paragraph and caption of Figure 4 were modified to clarify the experimental procedure:

Finally, an XRD pattern was collected at room temperature at conditions identical to those at the beginning of the experiment, after being irradiated by the X-ray beam for 200 s in total. A comparison of the XRD patterns at the beginning and end of the experiment is shown in Figure 4a.

Figure 4. (a) X-ray diffraction (XRD) patterns collected at room temperature at the beginning and end of the experimental session after 200 s of beam exposure. (b) Time evolution of *in situ* XRD patterns (lower image) during thermal cycles between 25 °C and 180 °C at heating and cooling rates of 1000 K s^{-1} (upper image).

The following paragraphs were added in the manuscript:

From the literature, it is known that several processes are involved in the radiation damage of organic molecules, such as formation of hydrogen (42) and other gasses (43), which leads to radicalization of the compound (44,45). The higher molecular stability at lower temperatures (42) indicates the important role of molecular mobility in radiation damage. We assume that in the crystalline and liquid crystalline (i.e. smectic B and nematic) states, the reduced molecular mobility leads to a higher probability of radical recombination. This generates less structural changes than in the isotropic liquid, where molecular mobility is high. Therefore, the sample is more prone to beam damage when irradiated in the isotropic liquid compared to the crystalline or liquid crystalline states.

To confirm the lack of thermal degradation of the sample due to a potentially absorbed thermal energy from the X-ray beam, a stability estimation measurement was performed *ex situ* via FDSC (46). The experimental details and results are given in the Supporting Information. The measurements reveal that the sample is resistant to thermal damage up to 320 °C in thermal pulses of 5 ms, where only sample evaporation is observed. The synchrotron X-ray pulses are nine orders of magnitude shorter and thus the thermal stability of the compound is expected to be higher at such short time scales. As no sample evaporation was observed during the *in situ* synchrotron experiments, the temperature increase due to the X-ray pulses must be lower than the exposure temperature in the stability estimation experiment. We thus conclude that the intensity decay in the XRD pattern can only result from a sample degradation due to radiation damage.

Additionally, the following changes in the conclusions were made:

For the setup used we found for the organic compound BCH-52 a small change of the average temperature by 0.2 K due to the temperature control of the DSC, an absorbed energy of 0.022 mW (approximately 220 W g^{-1}), and a significant beam damage after 0.55 s total exposure time including ca. 0.1 s at 180 °C in the isotropic liquid state. The absorbed energy is small compared to the heat-flow signal of the smectic B to nematic phase transformation in BCH-52. This indicates that the sample is not damaged by the thermal effect of the X-ray beam, but by radiation damage that generates structural changes in the sample. This has also been confirmed by thermal stability measurements described in the Supporting Information. The interlayer spacing of the smectic B phase is affected more than the intermolecular parameter of the compound, and the ordering of the liquid phase decreases upon beam exposure.

References (42-46) were added to the manuscript:

(42) Meents, A.; Gutmann, S.; Wagner A.; Schulze-Briese, C. Origin and temperature dependence of radiation damage in biological samples at cryogenic temperatures. *PNAS.* **2010**, *107*, 1094-1099

(43) Zubavichus, Y.; Fuchs, O.; Weinhardt, L.; Heske, C.; Umbach, E.; Denlinger, J.D.; Grunze, M. Soft X-Ray-Induced Decomposition of Amino Acids: An XPS, Mass Spectrometry, and NEXAFS Study. *Radiat. Res.* **2004**, *161*, 346–358

(44) Sutton, K. A.; Black, P. J.; Mercer, K. R.; Garman, E. F.; Owen, R. L.; Snell, E. H.; Bernhard, W. A. Insights into the mechanism of X-ray-induced disulfide-bond cleavage in lysozyme crystals based on EPR, optical absorption and X-ray diffraction studies. *Acta Crystallogr. D Biol. Crystallogr.* **2013**, *69*(12), 2381-2394

(45) Tashmetov, M. Y.; Ismatov, N. B.; Demidov, S. V.; Allayarov, S. R. X-Ray Photoelectron Spectroscopy and Raman Spectroscopy Studies of the Gamma-Irradiated Ethylene–Tetrafluoroethylene Copolymer. *High Energy Chem.* **2024**, *58*(2), 230-235.

(46) Schawe, J. E. K.; Ziegelmeier, S. Determination of the thermal short time stability of polymers by fast scanning calorimetry. *Thermochim. Acta*, **2016**, *623*, 80–85

Finally, the following section with references was added in the Supporting information document:

THERMAL STABILITY OF BCH-52

In order to evaluate the thermal stability of BCH-52 by potential heating due to absorption of the picosecond synchrotron X-ray pulses, we deployed a calorimetric technique originally developed to study the short-time stability of organic materials (1). For such studies, the Flash DSC 2+ with a UFH 1 sensor was used and purged with Ar at a flow rate of 40 ml min^{-1}. As shown in Figure S3(a), the temperature program consists of a sequence of analysis steps between 25 °C and 180 °C, equivalent to the *in situ* XRD experiments performed, plus thermal stress exposure steps between 180 °C and an elevated temperature T_i, where T_i is sequentially increased up to 320 °C in 10 °C steps. The thermal stress exposure steps were performed with heating and cooling ramps of 10,000 K s^{-1} and isothermal steps of Δt = 5 ms in between.

The heat flow curves of the sample are shown in the inset to Fig. S3(b) and were recorded during heating up to 180 °C at a rate of 1000 K s^{-1} (analysis step) after the various thermal stress exposure steps. As can be seen in Fig. S3(b), the temperature and shape of the transformation peaks remained unchanged, and only the peak area and heat flow of the baseline decreased. This indicates a mass loss due to some evaporation in the liquid state, but no further damage to the sample.

For quantitative analysis, we defined a stability parameter, $\Sigma_{H/C}$, obtained by dividing the enthalpy of the transformation peak at 155 °C by the heat capacity of the sample before the transformation. Figure S3(b) shows $\Sigma_{H/C}$ as a function of exposure temperature T_i, and illustrates that the stability parameter does not change with increasing T_i. In fact, the intensity of the transformation peak and the heat capacity decrease with continuous sample evaporation, but no thermal degradation is observed, even when overheating to 140 K above the maximum temperature of the *in situ* synchrotron X-ray experiment. This illustrates that thermal decomposition of the sample by its potential heating due to absorption of the picosecond X-ray pulses is unlikely.

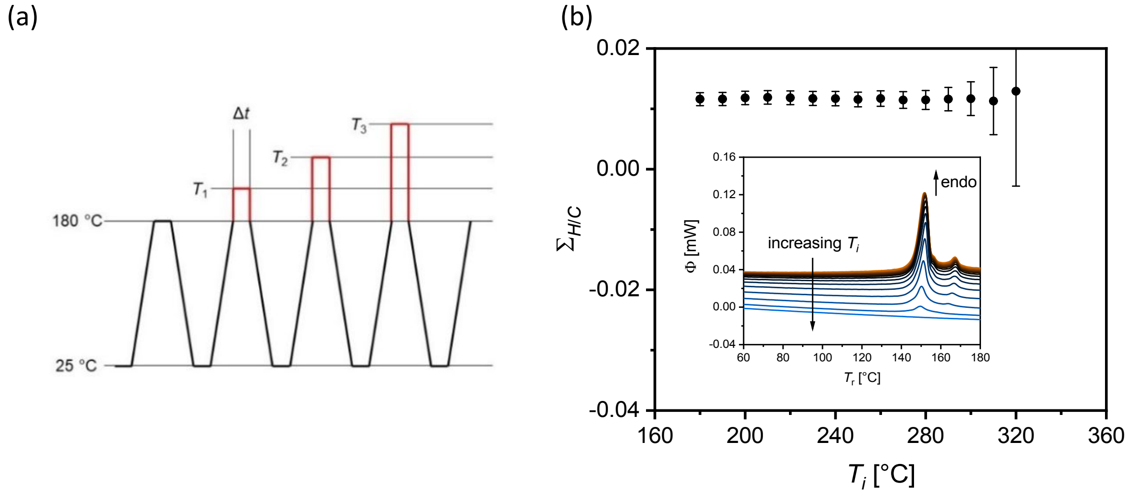

Figure S3. (a) Temperature program consisting of a sequence of analysis steps between 25 °C and 180 °C, plus thermal stress exposure steps between 180 °C and T_i, where T_i is sequentially increased up to 320 °C in 10 °C steps. (b) Heating curves of BCH-52 (inset) measured at a rate of 1000 K s^{-1}, and stability parameter, $\Sigma_{H/C}$, as a function of overheating temperature T_i. The error bars were determined from the uncertainties of measured heat capacity before the transformation at around 155 °C.

REFERENCES

(1) Schawe, J. E. K.; Ziegelmeier, S. Determination of the thermal short time stability of polymers by fast scanning calorimetry. *Thermochim. Acta*, **2016**, *623*, 80–85

Thank you for your consideration.

Yours sincerely, on behalf of all authors,

Štefan Stanko
Department of Materials
ETH Zurich
